# Diosgenin hemihydrate

**DOI:** 10.1107/S1600536812027912

**Published:** 2012-07-07

**Authors:** María-Guadalupe Hernández Linares, Sylvain Bernès, Marcos Flores-Alamo, Gabriel Guerrero-Luna, Anselmo A. Martínez-Gallegos

**Affiliations:** a Escuelas de Ingeniería en Petróleos e Ingeniería Química, Universidad del Istmo, Ciudad Universitaria s/n, 70760 Sto. Domingo Tehuantepec, Oax. 70760, Mexico; b DEP Facultad de Ciencias Químicas, UANL, Guerrero y Progreso S/N, Col. Treviño, 64570 Monterrey, NL, Mexico; c Facultad de Química, Universidad Nacional Autónoma de México, México DF 04510, Mexico

## Abstract

Diosgenin [or (22*R*,25*R*)-spirost-5-en-3β-ol] is the starting material of the Marker degradation, a cheap semi-synthesis of progesterone, which has been designated as an Inter­national Historic Chemical Landmark. Thus far, a single X-ray structure for diosgenin is known, namely its dimethyl sulfoxide solvate [Zhang *et al.* (2005 ▶). *Acta Cryst.* E**61**, o2324–o2325]. We have now determined the structure of the hemihydrate, C_27_H_42_O_3_·0.5H_2_O. The asymmetric unit contains two diosgenin mol­ecules, with quite similar conformations, and one water mol­ecule. Hy­droxy groups in steroids and water mol­ecules form O—H⋯O hydrogen-bonded *R*_5_^4^(10) ring motifs. Fused edge-sharing *R*(10) rings form a backbone oriented along [100], which aggregates the diosgenin mol­ecules in the crystal structure.

## Related literature

For historical background to the use of diosgenin in the synthesis of progesterone, see: Lehmann (1992 ▶); Djerassi (1992 ▶); Zhang *et al.* (2011 ▶). For the solubility of diosgenin, see: Chen *et al.* (2012 ▶). For the structure of diosgenin dimethyl sulfoxide solvate, see: Zhang *et al.* (2005 ▶). For a steroidal crystal structure featuring an 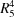
(10)-based supra­molecular structure, see: Xia *et al.* (2005 ▶).
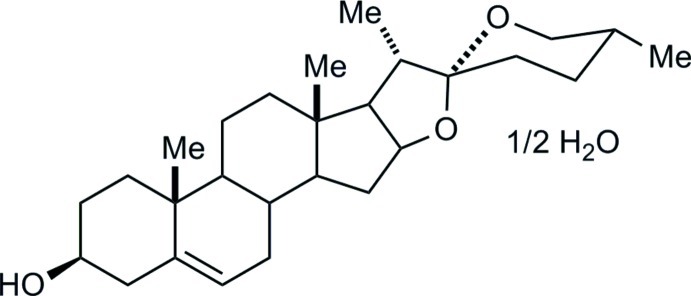


## Experimental

### 

#### Crystal data

C_27_H_42_O_3_·0.5H_2_O
*M* *_r_* = 423.61
Orthorhombic, 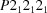

*a* = 7.3483 (5) Å
*b* = 19.698 (2) Å
*c* = 33.440 (3) Å
*V* = 4840.3 (8) Å^3^
*Z* = 8
Cu *K*α radiation
μ = 0.58 mm^−1^
*T* = 136 K
0.50 × 0.17 × 0.03 mm

#### Data collection

Oxford Diffraction Xcalibur Atlas Gemini diffractometer
Absorption correction: analytical [*CrysAlis PRO* (Oxford Diffraction, 2009 ▶); based on expressions derived by Clark & Reid (1995 ▶)] *T*_min_ = 0.821, *T*_max_ = 0.981
17450 measured reflections
4918 independent reflections
3573 reflections with *I* > 2σ(*I*)
*R*_int_ = 0.131

#### Refinement

*R*[*F* ^2^ > 2σ(*F* ^2^)] = 0.072
*wR*(*F* ^2^) = 0.177
*S* = 1.13
4918 reflections
570 parameters
3 restraints
H atoms treated by a mixture of independent and constrained refinement
Δρ_max_ = 0.31 e Å^−3^
Δρ_min_ = −0.36 e Å^−3^

### 

Data collection: *CrysAlis CCD* (Oxford Diffraction, 2009 ▶); cell refinement: *CrysAlis CCD*; data reduction: *CrysAlis RED* (Oxford Diffraction, 2009 ▶); program(s) used to solve structure: *SHELXS97* (Sheldrick, 2008 ▶); program(s) used to refine structure: *SHELXL97* (Sheldrick, 2008 ▶); molecular graphics: *SHELXTL* (Sheldrick, 2008 ▶) and *Mercury* (Macrae *et al.*, 2008 ▶); software used to prepare material for publication: *SHELXL97*.

## Supplementary Material

Crystal structure: contains datablock(s) I, global. DOI: 10.1107/S1600536812027912/gg2085sup1.cif

Structure factors: contains datablock(s) I. DOI: 10.1107/S1600536812027912/gg2085Isup2.hkl

Additional supplementary materials:  crystallographic information; 3D view; checkCIF report

## Figures and Tables

**Table 1:** Hydrogen-bond geometry (Å, °)

*D*—H⋯*A*	*D*—H	H⋯*A*	*D*⋯*A*	*D*—H⋯*A*
O1*W*—H2*W*⋯O3^i^	0.87 (2)	2.01 (2)	2.873 (6)	175 (6)
O1*W*—H1*W*⋯O53^i^	0.87 (2)	2.17 (3)	3.028 (6)	169 (5)
O3—H3⋯O1*W*	0.90 (7)	1.93 (7)	2.812 (6)	167 (6)
O53—H53⋯O3^i^	0.93 (7)	2.00 (7)	2.881 (6)	158 (6)

Symmetry code: (i) 
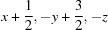
.

**Fig. 1.**
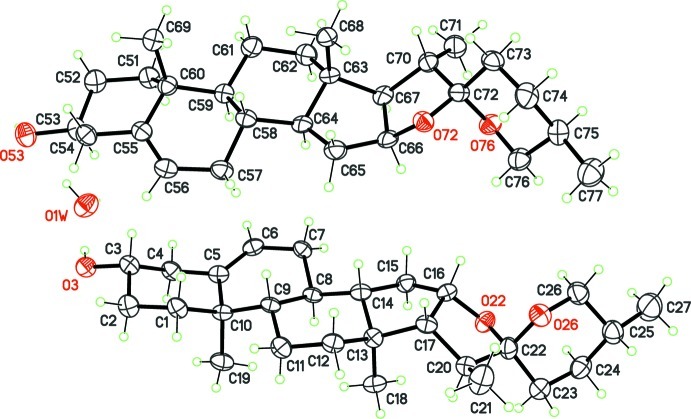
The asymmetric unit of the title compound. Displacement ellipsoids for non-H atoms are drawn at the 50% probability level.

**Fig. 2.**
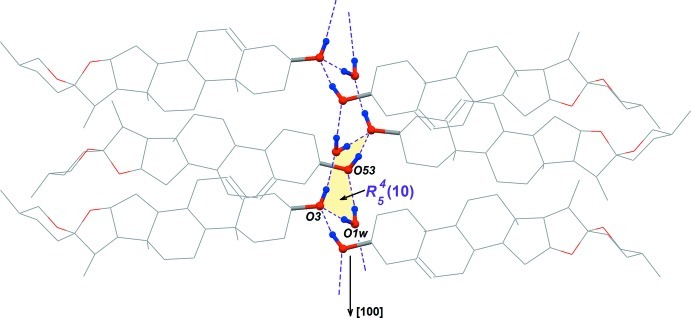
A part of the one-dimensional supramolecular structure of the title compound, based on six diosgenin and three water molecules. H atoms not involved in hydrogen bonds have been omitted, and the projection is normal to [010]. One R(10) motif is shown as a solid yellow polygon, with H-bonds components of the edges displayed as dashed bonds.

## supplementary crystallographic information

### Comment

Diosgenin is a steroid sapogenin available from natural sources, which is used for the commercial synthesis of steroid products like cortisone, pregnenolone and progesterone, amongst others (Djerassi, 1992; Zhang *et al.*, 2011). Its most significant application has been as a precursor in an economical semi-synthesis of progesterone, developed by Marker before World War II. This route, known as the Marker degradation (Lehmann, 1992), has been designated as an International Historic Chemical Landmark by the American Chemical Society and the *Sociedad Química de México*. However, the X-ray structure of diosgenin remains unknown, and only the dimethyl sulfoxide solvate has been characterized crystallographically so far (Zhang *et al.*, 2005). This is not surprising, taking into account the poor solubility of this steroid in polar solvents (Chen *et al.*, 2012).

We have now crystallized diosgenin hemihydrate. The asymmetric unit contains two diosgenin molecules and one lattice water molecule (Fig. 1). Diosgenin displays a rigid conformation, as reflected by the small r.m.s. deviation for the fit between independent molecules, of 0.16 Å. This conformation is also very close to that observed in the DMSO solvate (Zhang *et al.*, 2005; with r.m.s. deviations with the molecules of the title crystal: 0.22 and 0.23 Å).

In contrast with the DMSO solvate, in which discrete hydrogen bonds are formed between the steroid and the solvent, the hemihydrate gives rise to a supramolecular structure. Ring motifs *R*_5_^4^(10) are formed by three diosgenin and two water molecules. These motifs share edges with neighboring symmetry-related *R*(10) rings, forming a chain of fused rings in the crystal (Fig. 2), oriented in the [100] direction. This backbone based on efficient hydrogen bonds aggregates molecules in the crystal, and allows the crystallization of the hemihydrate. Such one-dimensional supramolecular structure is found in other steroids hydrates. Indeed, 11 identical supramolecular arrangements were found in the CSD, predominantly for androstane and androstene derivatives hydrates (*e.g*. Xia *et al.*, 2005). It thus seems that these kind of steroids functionalized with an alcohol group at C3 should have a propensity to crystallize as hydrates, since a stabilizing supramolecular structure may be arranged.

### Experimental

Diosgenin hemihydrate was initially obtained as unreacted material in a reaction attempt between diosgenin and terephthaloyl chloride. The same hemihydrate may be obtained by stirring diosgenin in CH_2_Cl_2_ (1 mmol in 20 ml) until complete dissolution. After washing the solution with distilled water, the organic phase is dried with anhydrous Na_2_SO_4_, filtered, and concentrated under reduced pressure. The residue is then crystallized from hexane/acetone (3:2).

### Refinement

The search for a single-crystal was challenging. After a number of attempts, a sample with a thickness limited to 0.03 mm was collected, although data reduction revealed that it was not a single-crystal. The poor sample quality is reflected in the high residual for symmetry-equivalent reflections, *R*_int_ = 0.131. Hydroxy H atoms, H3, H53, H1W and H2W, were found in a difference map and refined freely. The geometry for the water molecule was however restrained, with O—H = 0.85 (2) Å, and H1W···H2W = 1.34 (1) Å. Other H atoms, bonded to C atoms, were placed in idealized positions and refined as riding to their carrier atoms. Isotropic displacement parameters for H atoms were calculated as *U*_iso_(H) = *xU*_eq_(carrier atom) where *x* = 1.5 for methyl H atoms and *x* = 1.2 for other H atoms. The absolute configuration was assigned from chiral centers with known configuration in the steroidal nucleus, and measured Friedel pairs (3683) were merged.

### Crystal data

**Table:**

C_27_H_42_O_3_·0.5H_2_O	*D*_x_ = 1.163 Mg m^−^^3^
*M**_r_* = 423.61	Melting point: 590 K
Orthorhombic, *P*2_1_2_1_2_1_	Cu *K*α radiation, λ = 1.54180 Å
Hall symbol: P 2ac 2ab	Cell parameters from 1880 reflections
*a* = 7.3483 (5) Å	θ = 3.5–67.7°
*b* = 19.698 (2) Å	µ = 0.58 mm^−^^1^
*c* = 33.440 (3) Å	*T* = 136 K
*V* = 4840.3 (8) Å^3^	Plate, colourless
*Z* = 8	0.50 × 0.17 × 0.03 mm
*F*(000) = 1864	

### Data collection

**Table:**

Oxford Diffraction Xcalibur Atlas Gemini diffractometer	4918 independent reflections
Radiation source: Enhance (Cu) X-ray Source	3573 reflections with *I* > 2σ(*I*)
Graphite monochromator	*R*_int_ = 0.131
Detector resolution: 10.4685 pixels mm^-1^	θ_max_ = 67.4°, θ_min_ = 3.5°
ω scans	*h* = −5→8
Absorption correction: analytical [*CrysAlis PRO* (Oxford Diffraction, 2009); based on expressions derived by Clark & Reid (1995)]	*k* = −20→23
*T*_min_ = 0.821, *T*_max_ = 0.981	*l* = −40→34
17450 measured reflections	

### Refinement

**Table:**

Refinement on *F*^2^	Primary atom site location: structure-invariant direct methods
Least-squares matrix: full	Secondary atom site location: difference Fourier map
*R*[*F*^2^ > 2σ(*F*^2^)] = 0.072	Hydrogen site location: inferred from neighbouring sites
*wR*(*F*^2^) = 0.177	H atoms treated by a mixture of independent and constrained refinement
*S* = 1.13	*w* = 1/[σ^2^(*F*_o_^2^) + (0.05*P*)^2^] where *P* = (*F*_o_^2^ + 2*F*_c_^2^)/3
4918 reflections	(Δ/σ)_max_ < 0.001
570 parameters	Δρ_max_ = 0.31 e Å^−^^3^
3 restraints	Δρ_min_ = −0.36 e Å^−^^3^
0 constraints	

### Fractional atomic coordinates and isotropic or equivalent isotropic displacement parameters (Å^2^)

**Table:**

	*x*	*y*	*z*	*U*_iso_*/*U*_eq_	
C1	−0.5923 (7)	0.7342 (3)	0.13594 (14)	0.0290 (12)	
H1A	−0.7238	0.7308	0.1421	0.035*	
H1B	−0.5541	0.7815	0.1413	0.035*	
C2	−0.5635 (7)	0.7189 (3)	0.09132 (15)	0.0334 (13)	
H2A	−0.6112	0.6731	0.0851	0.040*	
H2B	−0.6317	0.7523	0.0750	0.040*	
C3	−0.3633 (7)	0.7222 (3)	0.08070 (15)	0.0289 (11)	
H3A	−0.3172	0.7694	0.0851	0.035*	
O3	−0.3447 (5)	0.7044 (2)	0.03903 (10)	0.0320 (8)	
H3	−0.233 (9)	0.695 (3)	0.0297 (17)	0.038*	
C4	−0.2557 (7)	0.6725 (3)	0.10695 (15)	0.0308 (12)	
H4A	−0.2920	0.6254	0.1002	0.037*	
H4B	−0.1243	0.6773	0.1011	0.037*	
C5	−0.2877 (7)	0.6847 (3)	0.15135 (15)	0.0272 (11)	
C6	−0.1487 (7)	0.6917 (3)	0.17587 (15)	0.0279 (11)	
H6A	−0.0298	0.6915	0.1647	0.033*	
C7	−0.1669 (7)	0.7001 (3)	0.22032 (14)	0.0259 (11)	
H7A	−0.1392	0.7477	0.2275	0.031*	
H7B	−0.0762	0.6707	0.2338	0.031*	
C8	−0.3565 (7)	0.6821 (2)	0.23572 (14)	0.0237 (11)	
H8A	−0.3715	0.6316	0.2348	0.028*	
C9	−0.4999 (7)	0.7147 (2)	0.20819 (14)	0.0231 (10)	
H9A	−0.4682	0.7640	0.2064	0.028*	
C10	−0.4859 (7)	0.6858 (2)	0.16451 (15)	0.0248 (11)	
C11	−0.6944 (7)	0.7113 (3)	0.22522 (15)	0.0310 (12)	
H11A	−0.7748	0.7406	0.2089	0.037*	
H11B	−0.7397	0.6641	0.2229	0.037*	
C12	−0.7067 (7)	0.7334 (3)	0.26892 (15)	0.0284 (11)	
H12A	−0.8326	0.7265	0.2787	0.034*	
H12B	−0.6780	0.7824	0.2710	0.034*	
C13	−0.5748 (7)	0.6930 (3)	0.29506 (14)	0.0257 (11)	
C14	−0.3842 (7)	0.7060 (2)	0.27863 (14)	0.0249 (11)	
H14A	−0.3683	0.7564	0.2782	0.030*	
C15	−0.2581 (7)	0.6793 (3)	0.31154 (14)	0.0289 (11)	
H15A	−0.2504	0.6291	0.3111	0.035*	
H15B	−0.1342	0.6986	0.3091	0.035*	
C16	−0.3531 (7)	0.7045 (3)	0.34912 (15)	0.0274 (11)	
H16A	−0.2916	0.7462	0.3595	0.033*	
C17	−0.5543 (7)	0.7201 (2)	0.33852 (15)	0.0263 (11)	
H17A	−0.5735	0.7704	0.3386	0.032*	
C18	−0.6231 (8)	0.6166 (3)	0.29475 (16)	0.0329 (12)	
H18A	−0.6083	0.5985	0.2676	0.049*	
H18B	−0.7496	0.6106	0.3034	0.049*	
H18C	−0.5422	0.5922	0.3131	0.049*	
C19	−0.5637 (8)	0.6130 (3)	0.16203 (16)	0.0368 (13)	
H19A	−0.5277	0.5923	0.1366	0.055*	
H19B	−0.6968	0.6148	0.1637	0.055*	
H19C	−0.5160	0.5859	0.1842	0.055*	
C20	−0.6654 (8)	0.6881 (3)	0.37263 (15)	0.0331 (12)	
H20A	−0.7202	0.6451	0.3622	0.040*	
C21	−0.8195 (9)	0.7317 (4)	0.38853 (18)	0.0487 (16)	
H21A	−0.8803	0.7080	0.4106	0.073*	
H21B	−0.9073	0.7404	0.3671	0.073*	
H21C	−0.7703	0.7750	0.3982	0.073*	
C22	−0.5214 (8)	0.6686 (3)	0.40287 (14)	0.0314 (12)	
O22	−0.3637 (5)	0.65326 (18)	0.38008 (10)	0.0306 (8)	
C23	−0.5652 (9)	0.6065 (3)	0.42891 (16)	0.0375 (13)	
H23A	−0.6838	0.6135	0.4423	0.045*	
H23B	−0.5756	0.5658	0.4117	0.045*	
C24	−0.4197 (9)	0.5945 (3)	0.46013 (16)	0.0382 (13)	
H24A	−0.3055	0.5804	0.4468	0.046*	
H24B	−0.4584	0.5573	0.4781	0.046*	
C25	−0.3849 (9)	0.6590 (3)	0.48480 (16)	0.0388 (14)	
H25A	−0.4967	0.6702	0.5005	0.047*	
C26	−0.3448 (8)	0.7168 (3)	0.45564 (16)	0.0375 (13)	
H26A	−0.3265	0.7594	0.4709	0.045*	
H26B	−0.2306	0.7068	0.4411	0.045*	
O26	−0.4881 (6)	0.72624 (19)	0.42763 (10)	0.0343 (9)	
C27	−0.2262 (10)	0.6504 (5)	0.5131 (2)	0.062 (2)	
H27A	−0.1991	0.6939	0.5260	0.094*	
H27B	−0.1193	0.6349	0.4982	0.094*	
H27C	−0.2574	0.6167	0.5336	0.094*	
C51	0.1484 (8)	0.9123 (3)	0.08829 (15)	0.0332 (12)	
H51A	0.2659	0.9356	0.0929	0.040*	
H51B	0.1635	0.8644	0.0966	0.040*	
C52	0.1074 (8)	0.9138 (3)	0.04339 (16)	0.0380 (14)	
H52A	0.1088	0.9614	0.0338	0.046*	
H52B	0.2026	0.8884	0.0288	0.046*	
C53	−0.0776 (8)	0.8824 (3)	0.03508 (15)	0.0346 (13)	
H53A	−0.0774	0.8345	0.0450	0.041*	
O53	−0.1234 (6)	0.8824 (2)	−0.00652 (11)	0.0398 (10)	
H53	−0.030 (10)	0.864 (4)	−0.0216 (18)	0.048*	
C54	−0.2235 (8)	0.9220 (3)	0.05734 (15)	0.0326 (12)	
H54A	−0.2259	0.9694	0.0475	0.039*	
H54B	−0.3441	0.9015	0.0520	0.039*	
C55	−0.1871 (8)	0.9218 (3)	0.10181 (15)	0.0284 (11)	
C56	−0.3132 (7)	0.8993 (3)	0.12685 (16)	0.0307 (12)	
H56A	−0.4271	0.8861	0.1159	0.037*	
C57	−0.2893 (8)	0.8931 (3)	0.17109 (16)	0.0330 (12)	
H57A	−0.2695	0.8448	0.1780	0.040*	
H57B	−0.4024	0.9082	0.1845	0.040*	
C58	−0.1290 (7)	0.9352 (2)	0.18683 (15)	0.0265 (11)	
H58A	−0.1632	0.9843	0.1866	0.032*	
C59	0.0371 (7)	0.9245 (3)	0.15971 (14)	0.0255 (11)	
H59A	0.0596	0.8745	0.1592	0.031*	
C60	0.0013 (8)	0.9458 (3)	0.11552 (15)	0.0294 (12)	
C61	0.2116 (8)	0.9569 (3)	0.17732 (16)	0.0346 (13)	
H61A	0.3171	0.9434	0.1607	0.042*	
H61B	0.2006	1.0069	0.1760	0.042*	
C62	0.2473 (8)	0.9358 (3)	0.22073 (16)	0.0353 (13)	
H62A	0.2749	0.8867	0.2217	0.042*	
H62B	0.3549	0.9606	0.2309	0.042*	
C63	0.0844 (7)	0.9507 (3)	0.24752 (15)	0.0257 (11)	
C64	−0.0824 (7)	0.9140 (2)	0.22941 (14)	0.0258 (11)	
H64A	−0.0484	0.8650	0.2279	0.031*	
C65	−0.2273 (8)	0.9192 (3)	0.26234 (15)	0.0345 (13)	
H65A	−0.2837	0.9648	0.2629	0.041*	
H65B	−0.3235	0.8845	0.2588	0.041*	
C66	−0.1155 (8)	0.9059 (3)	0.29997 (15)	0.0299 (12)	
H66A	−0.1350	0.8584	0.3096	0.036*	
C67	0.0877 (8)	0.9178 (3)	0.29005 (14)	0.0268 (11)	
H67A	0.1514	0.8730	0.2886	0.032*	
C68	0.0511 (8)	1.0276 (3)	0.25050 (15)	0.0319 (12)	
H68A	0.0178	1.0454	0.2241	0.048*	
H68B	0.1622	1.0501	0.2599	0.048*	
H68C	−0.0480	1.0364	0.2694	0.048*	
C69	0.0099 (9)	1.0231 (3)	0.11037 (16)	0.0367 (13)	
H69A	−0.0763	1.0446	0.1288	0.055*	
H69B	−0.0218	1.0349	0.0828	0.055*	
H69C	0.1334	1.0390	0.1163	0.055*	
C70	0.1610 (8)	0.9579 (3)	0.32608 (15)	0.0291 (12)	
H70A	0.1786	1.0060	0.3173	0.035*	
C71	0.3431 (8)	0.9323 (3)	0.34269 (17)	0.0397 (14)	
H71A	0.4351	0.9332	0.3215	0.060*	
H71B	0.3286	0.8857	0.3525	0.060*	
H71C	0.3821	0.9617	0.3647	0.060*	
C72	0.0016 (8)	0.9564 (3)	0.35482 (15)	0.0309 (12)	
O72	−0.1563 (5)	0.95371 (18)	0.33112 (10)	0.0299 (8)	
C73	−0.0145 (8)	1.0185 (3)	0.38266 (15)	0.0329 (12)	
H73A	0.1006	1.0242	0.3977	0.039*	
H73B	−0.0338	1.0598	0.3664	0.039*	
C74	−0.1697 (9)	1.0106 (3)	0.41173 (16)	0.0380 (13)	
H74A	−0.2865	1.0105	0.3970	0.046*	
H74B	−0.1708	1.0495	0.4305	0.046*	
C75	−0.1508 (9)	0.9442 (3)	0.43536 (15)	0.0360 (13)	
H75A	−0.0380	0.9465	0.4521	0.043*	
C76	−0.1306 (9)	0.8864 (3)	0.40496 (16)	0.0360 (13)	
H76A	−0.1138	0.8430	0.4195	0.043*	
H76B	−0.2439	0.8828	0.3891	0.043*	
O76	0.0191 (5)	0.89658 (17)	0.37864 (10)	0.0321 (8)	
C77	−0.3121 (10)	0.9321 (3)	0.46238 (19)	0.0493 (16)	
H77A	−0.3322	0.9722	0.4791	0.074*	
H77B	−0.2882	0.8926	0.4795	0.074*	
H77C	−0.4206	0.9235	0.4461	0.074*	
O1W	0.0209 (6)	0.6938 (2)	0.01496 (13)	0.0484 (11)	
H1W	0.118 (6)	0.669 (3)	0.015 (2)	0.058*	
H2W	0.055 (8)	0.725 (2)	−0.0017 (17)	0.058*	

### Atomic displacement parameters (Å^2^)

**Table:**

	*U*^11^	*U*^22^	*U*^33^	*U*^12^	*U*^13^	*U*^23^
C1	0.019 (3)	0.036 (3)	0.032 (3)	0.005 (2)	−0.001 (2)	−0.004 (2)
C2	0.026 (3)	0.039 (3)	0.035 (3)	0.008 (3)	0.000 (2)	−0.002 (2)
C3	0.027 (3)	0.027 (3)	0.033 (3)	0.002 (2)	−0.002 (2)	−0.005 (2)
O3	0.026 (2)	0.039 (2)	0.0305 (18)	0.0009 (18)	0.0054 (17)	−0.0040 (15)
C4	0.022 (3)	0.036 (3)	0.034 (3)	0.003 (2)	−0.001 (2)	−0.004 (2)
C5	0.022 (3)	0.024 (3)	0.035 (3)	0.002 (2)	0.001 (2)	−0.001 (2)
C6	0.020 (3)	0.027 (3)	0.036 (3)	0.002 (2)	0.008 (2)	0.002 (2)
C7	0.019 (3)	0.029 (3)	0.030 (2)	0.001 (2)	−0.002 (2)	−0.0029 (19)
C8	0.024 (3)	0.016 (2)	0.031 (2)	−0.002 (2)	−0.001 (2)	−0.0033 (18)
C9	0.022 (2)	0.016 (2)	0.031 (2)	−0.001 (2)	0.000 (2)	−0.0039 (17)
C10	0.019 (3)	0.019 (2)	0.037 (3)	0.001 (2)	0.000 (2)	0.0010 (19)
C11	0.028 (3)	0.032 (3)	0.034 (3)	0.004 (2)	−0.004 (2)	−0.001 (2)
C12	0.017 (3)	0.033 (3)	0.036 (3)	0.003 (2)	0.002 (2)	−0.001 (2)
C13	0.024 (3)	0.024 (3)	0.029 (2)	0.005 (2)	−0.001 (2)	0.0004 (19)
C14	0.026 (3)	0.014 (2)	0.035 (3)	0.002 (2)	0.002 (2)	−0.0013 (18)
C15	0.026 (3)	0.030 (3)	0.031 (3)	−0.001 (2)	0.001 (2)	0.0035 (19)
C16	0.028 (3)	0.023 (3)	0.031 (2)	−0.002 (2)	0.003 (2)	0.0028 (19)
C17	0.028 (3)	0.017 (2)	0.034 (3)	0.001 (2)	0.000 (2)	−0.0029 (19)
C18	0.029 (3)	0.027 (3)	0.042 (3)	−0.004 (2)	0.002 (3)	0.003 (2)
C19	0.040 (3)	0.026 (3)	0.045 (3)	−0.009 (3)	−0.002 (3)	−0.007 (2)
C20	0.031 (3)	0.036 (3)	0.032 (3)	−0.006 (3)	0.000 (2)	−0.001 (2)
C21	0.033 (3)	0.070 (5)	0.043 (3)	0.015 (3)	0.004 (3)	0.009 (3)
C22	0.034 (3)	0.036 (3)	0.024 (2)	0.001 (3)	0.002 (2)	−0.003 (2)
O22	0.030 (2)	0.031 (2)	0.0306 (18)	0.0057 (17)	0.0020 (17)	0.0077 (14)
C23	0.041 (3)	0.038 (3)	0.034 (3)	−0.005 (3)	0.000 (3)	0.003 (2)
C24	0.045 (3)	0.034 (3)	0.035 (3)	0.001 (3)	0.006 (3)	0.004 (2)
C25	0.036 (3)	0.046 (3)	0.034 (3)	0.002 (3)	−0.003 (3)	0.000 (2)
C26	0.035 (3)	0.043 (3)	0.035 (3)	0.000 (3)	0.000 (3)	−0.006 (2)
O26	0.036 (2)	0.031 (2)	0.0358 (18)	0.0032 (18)	−0.0001 (18)	−0.0039 (15)
C27	0.045 (4)	0.092 (6)	0.050 (4)	−0.005 (4)	−0.006 (4)	0.010 (4)
C51	0.024 (3)	0.041 (3)	0.035 (3)	0.004 (3)	0.003 (2)	−0.002 (2)
C52	0.032 (3)	0.045 (3)	0.036 (3)	0.004 (3)	0.005 (3)	−0.001 (2)
C53	0.036 (3)	0.038 (3)	0.030 (3)	0.004 (3)	−0.005 (3)	0.000 (2)
O53	0.043 (3)	0.044 (2)	0.0317 (19)	0.003 (2)	−0.0002 (19)	−0.0063 (16)
C54	0.026 (3)	0.036 (3)	0.036 (3)	0.002 (3)	−0.003 (2)	0.000 (2)
C55	0.030 (3)	0.020 (3)	0.036 (3)	0.004 (2)	−0.005 (2)	0.0000 (19)
C56	0.024 (3)	0.030 (3)	0.038 (3)	0.000 (2)	0.003 (2)	−0.006 (2)
C57	0.025 (3)	0.036 (3)	0.038 (3)	−0.004 (3)	0.001 (2)	−0.002 (2)
C58	0.026 (3)	0.019 (3)	0.034 (3)	0.002 (2)	−0.002 (2)	−0.0015 (18)
C59	0.023 (3)	0.022 (2)	0.031 (3)	0.006 (2)	−0.001 (2)	0.0004 (19)
C60	0.031 (3)	0.026 (3)	0.032 (2)	0.001 (2)	−0.001 (3)	0.0020 (19)
C61	0.027 (3)	0.040 (3)	0.037 (3)	0.000 (3)	0.002 (3)	0.004 (2)
C62	0.032 (3)	0.034 (3)	0.040 (3)	0.003 (3)	−0.004 (3)	0.002 (2)
C63	0.025 (3)	0.021 (3)	0.032 (2)	−0.003 (2)	0.002 (2)	0.0010 (19)
C64	0.029 (3)	0.014 (2)	0.035 (3)	0.002 (2)	−0.002 (2)	0.0031 (18)
C65	0.030 (3)	0.038 (3)	0.036 (3)	−0.008 (3)	0.004 (3)	−0.002 (2)
C66	0.039 (3)	0.019 (3)	0.032 (3)	−0.005 (2)	0.003 (3)	0.0017 (19)
C67	0.035 (3)	0.013 (2)	0.033 (3)	0.007 (2)	0.001 (2)	0.0033 (18)
C68	0.036 (3)	0.026 (3)	0.033 (3)	−0.003 (2)	−0.004 (3)	0.003 (2)
C69	0.040 (3)	0.037 (3)	0.033 (3)	−0.002 (3)	−0.003 (3)	0.003 (2)
C70	0.035 (3)	0.023 (2)	0.030 (3)	0.002 (2)	−0.002 (2)	0.0025 (19)
C71	0.031 (3)	0.049 (4)	0.039 (3)	−0.001 (3)	0.000 (3)	0.000 (2)
C72	0.031 (3)	0.029 (3)	0.033 (3)	−0.007 (3)	−0.002 (3)	0.002 (2)
O72	0.030 (2)	0.0288 (18)	0.0314 (18)	−0.0001 (17)	−0.0032 (17)	−0.0004 (14)
C73	0.038 (3)	0.024 (3)	0.037 (3)	−0.003 (3)	−0.005 (3)	0.000 (2)
C74	0.045 (3)	0.034 (3)	0.035 (3)	0.006 (3)	0.005 (3)	−0.007 (2)
C75	0.037 (3)	0.041 (3)	0.030 (3)	0.004 (3)	0.000 (3)	−0.001 (2)
C76	0.044 (4)	0.028 (3)	0.037 (3)	0.001 (3)	0.001 (3)	0.003 (2)
O76	0.037 (2)	0.0231 (18)	0.0366 (18)	0.0018 (18)	0.0021 (18)	0.0035 (14)
C77	0.054 (4)	0.049 (4)	0.045 (3)	0.002 (3)	0.007 (3)	0.001 (3)
O1W	0.038 (2)	0.053 (3)	0.053 (2)	0.005 (2)	0.010 (2)	0.009 (2)

### Geometric parameters (Å, º)

**Table:**

C1—C2	1.537 (7)	C51—C60	1.559 (8)
C1—C10	1.559 (7)	C51—H51A	0.9900
C1—H1A	0.9900	C51—H51B	0.9900
C1—H1B	0.9900	C52—C53	1.519 (8)
C2—C3	1.515 (8)	C52—H52A	0.9900
C2—H2A	0.9900	C52—H52B	0.9900
C2—H2B	0.9900	C53—O53	1.431 (6)
C3—O3	1.443 (6)	C53—C54	1.521 (8)
C3—C4	1.535 (7)	C53—H53A	1.0000
C3—H3A	1.0000	O53—H53	0.93 (7)
O3—H3	0.90 (7)	C54—C55	1.511 (7)
C4—C5	1.522 (7)	C54—H54A	0.9900
C4—H4A	0.9900	C54—H54B	0.9900
C4—H4B	0.9900	C55—C56	1.326 (8)
C5—C6	1.316 (8)	C55—C60	1.533 (8)
C5—C10	1.522 (7)	C56—C57	1.495 (7)
C6—C7	1.502 (7)	C56—H56A	0.9500
C6—H6A	0.9500	C57—C58	1.534 (7)
C7—C8	1.527 (7)	C57—H57A	0.9900
C7—H7A	0.9900	C57—H57B	0.9900
C7—H7B	0.9900	C58—C64	1.523 (7)
C8—C14	1.524 (7)	C58—C59	1.535 (7)
C8—C9	1.541 (7)	C58—H58A	1.0000
C8—H8A	1.0000	C59—C61	1.548 (7)
C9—C11	1.540 (7)	C59—C60	1.558 (7)
C9—C10	1.571 (6)	C59—H59A	1.0000
C9—H9A	1.0000	C60—C69	1.534 (8)
C10—C19	1.546 (7)	C61—C62	1.532 (7)
C11—C12	1.528 (7)	C61—H61A	0.9900
C11—H11A	0.9900	C61—H61B	0.9900
C11—H11B	0.9900	C62—C63	1.524 (8)
C12—C13	1.529 (7)	C62—H62A	0.9900
C12—H12A	0.9900	C62—H62B	0.9900
C12—H12B	0.9900	C63—C68	1.538 (8)
C13—C14	1.526 (7)	C63—C64	1.546 (7)
C13—C18	1.546 (8)	C63—C67	1.563 (7)
C13—C17	1.556 (7)	C64—C65	1.535 (7)
C14—C15	1.532 (7)	C64—H64A	1.0000
C14—H14A	1.0000	C65—C66	1.525 (7)
C15—C16	1.521 (7)	C65—H65A	0.9900
C15—H15A	0.9900	C65—H65B	0.9900
C15—H15B	0.9900	C66—O72	1.436 (6)
C16—O22	1.448 (6)	C66—C67	1.548 (8)
C16—C17	1.551 (7)	C66—H66A	1.0000
C16—H16A	1.0000	C67—C70	1.538 (7)
C17—C20	1.538 (7)	C67—H67A	1.0000
C17—H17A	1.0000	C68—H68A	0.9800
C18—H18A	0.9800	C68—H68B	0.9800
C18—H18B	0.9800	C68—H68C	0.9800
C18—H18C	0.9800	C69—H69A	0.9800
C19—H19A	0.9800	C69—H69B	0.9800
C19—H19B	0.9800	C69—H69C	0.9800
C19—H19C	0.9800	C70—C72	1.515 (8)
C20—C22	1.513 (8)	C70—C71	1.534 (8)
C20—C21	1.518 (8)	C70—H70A	1.0000
C20—H20A	1.0000	C71—H71A	0.9800
C21—H21A	0.9800	C71—H71B	0.9800
C21—H21B	0.9800	C71—H71C	0.9800
C21—H21C	0.9800	C72—O72	1.406 (7)
C22—O22	1.420 (7)	C72—O76	1.429 (6)
C22—O26	1.426 (6)	C72—C73	1.541 (7)
C22—C23	1.536 (8)	C73—C74	1.507 (8)
C23—C24	1.513 (8)	C73—H73A	0.9900
C23—H23A	0.9900	C73—H73B	0.9900
C23—H23B	0.9900	C74—C75	1.534 (8)
C24—C25	1.536 (8)	C74—H74A	0.9900
C24—H24A	0.9900	C74—H74B	0.9900
C24—H24B	0.9900	C75—C77	1.509 (9)
C25—C27	1.512 (9)	C75—C76	1.533 (8)
C25—C26	1.528 (8)	C75—H75A	1.0000
C25—H25A	1.0000	C76—O76	1.423 (7)
C26—O26	1.422 (7)	C76—H76A	0.9900
C26—H26A	0.9900	C76—H76B	0.9900
C26—H26B	0.9900	C77—H77A	0.9800
C27—H27A	0.9800	C77—H77B	0.9800
C27—H27B	0.9800	C77—H77C	0.9800
C27—H27C	0.9800	O1W—H1W	0.87 (2)
C51—C52	1.532 (7)	O1W—H2W	0.87 (2)
			
C2—C1—C10	114.0 (4)	C52—C51—H51A	108.4
C2—C1—H1A	108.8	C60—C51—H51A	108.4
C10—C1—H1A	108.8	C52—C51—H51B	108.4
C2—C1—H1B	108.8	C60—C51—H51B	108.4
C10—C1—H1B	108.8	H51A—C51—H51B	107.5
H1A—C1—H1B	107.7	C53—C52—C51	110.3 (5)
C3—C2—C1	110.7 (4)	C53—C52—H52A	109.6
C3—C2—H2A	109.5	C51—C52—H52A	109.6
C1—C2—H2A	109.5	C53—C52—H52B	109.6
C3—C2—H2B	109.5	C51—C52—H52B	109.6
C1—C2—H2B	109.5	H52A—C52—H52B	108.1
H2A—C2—H2B	108.1	O53—C53—C52	112.9 (5)
O3—C3—C2	107.9 (4)	O53—C53—C54	108.0 (5)
O3—C3—C4	110.4 (4)	C52—C53—C54	109.4 (5)
C2—C3—C4	109.8 (5)	O53—C53—H53A	108.8
O3—C3—H3A	109.6	C52—C53—H53A	108.8
C2—C3—H3A	109.6	C54—C53—H53A	108.8
C4—C3—H3A	109.6	C53—O53—H53	111 (4)
C3—O3—H3	118 (4)	C55—C54—C53	110.8 (5)
C5—C4—C3	112.2 (4)	C55—C54—H54A	109.5
C5—C4—H4A	109.2	C53—C54—H54A	109.5
C3—C4—H4A	109.2	C55—C54—H54B	109.5
C5—C4—H4B	109.2	C53—C54—H54B	109.5
C3—C4—H4B	109.2	H54A—C54—H54B	108.1
H4A—C4—H4B	107.9	C56—C55—C54	119.9 (5)
C6—C5—C10	124.1 (5)	C56—C55—C60	123.1 (5)
C6—C5—C4	120.3 (5)	C54—C55—C60	117.0 (5)
C10—C5—C4	115.6 (5)	C55—C56—C57	124.8 (5)
C5—C6—C7	124.0 (5)	C55—C56—H56A	117.6
C5—C6—H6A	118.0	C57—C56—H56A	117.6
C7—C6—H6A	118.0	C56—C57—C58	112.7 (5)
C6—C7—C8	112.9 (4)	C56—C57—H57A	109.1
C6—C7—H7A	109.0	C58—C57—H57A	109.1
C8—C7—H7A	109.0	C56—C57—H57B	109.1
C6—C7—H7B	109.0	C58—C57—H57B	109.1
C8—C7—H7B	109.0	H57A—C57—H57B	107.8
H7A—C7—H7B	107.8	C64—C58—C57	110.2 (4)
C14—C8—C7	111.6 (4)	C64—C58—C59	109.6 (4)
C14—C8—C9	110.0 (4)	C57—C58—C59	109.5 (4)
C7—C8—C9	109.0 (4)	C64—C58—H58A	109.1
C14—C8—H8A	108.7	C57—C58—H58A	109.1
C7—C8—H8A	108.7	C59—C58—H58A	109.1
C9—C8—H8A	108.7	C58—C59—C61	112.2 (4)
C11—C9—C8	113.3 (4)	C58—C59—C60	112.9 (4)
C11—C9—C10	112.9 (4)	C61—C59—C60	113.0 (4)
C8—C9—C10	111.0 (4)	C58—C59—H59A	106.0
C11—C9—H9A	106.3	C61—C59—H59A	106.0
C8—C9—H9A	106.3	C60—C59—H59A	106.0
C10—C9—H9A	106.3	C55—C60—C69	108.0 (5)
C5—C10—C19	108.9 (5)	C55—C60—C59	110.7 (4)
C5—C10—C1	108.2 (4)	C69—C60—C59	111.5 (4)
C19—C10—C1	110.4 (4)	C55—C60—C51	108.8 (4)
C5—C10—C9	109.7 (4)	C69—C60—C51	109.0 (5)
C19—C10—C9	111.2 (4)	C59—C60—C51	108.9 (4)
C1—C10—C9	108.4 (4)	C62—C61—C59	113.0 (5)
C12—C11—C9	113.3 (4)	C62—C61—H61A	109.0
C12—C11—H11A	108.9	C59—C61—H61A	109.0
C9—C11—H11A	108.9	C62—C61—H61B	109.0
C12—C11—H11B	108.9	C59—C61—H61B	109.0
C9—C11—H11B	108.9	H61A—C61—H61B	107.8
H11A—C11—H11B	107.7	C63—C62—C61	111.8 (5)
C11—C12—C13	111.1 (4)	C63—C62—H62A	109.3
C11—C12—H12A	109.4	C61—C62—H62A	109.3
C13—C12—H12A	109.4	C63—C62—H62B	109.3
C11—C12—H12B	109.4	C61—C62—H62B	109.3
C13—C12—H12B	109.4	H62A—C62—H62B	107.9
H12A—C12—H12B	108.0	C62—C63—C68	110.7 (4)
C14—C13—C12	106.8 (4)	C62—C63—C64	107.6 (4)
C14—C13—C18	111.8 (4)	C68—C63—C64	111.1 (5)
C12—C13—C18	110.9 (4)	C62—C63—C67	116.3 (5)
C14—C13—C17	101.0 (4)	C68—C63—C67	110.6 (4)
C12—C13—C17	114.6 (4)	C64—C63—C67	100.1 (4)
C18—C13—C17	111.3 (4)	C58—C64—C65	119.8 (5)
C8—C14—C13	114.2 (4)	C58—C64—C63	114.6 (4)
C8—C14—C15	119.3 (4)	C65—C64—C63	103.8 (4)
C13—C14—C15	103.8 (4)	C58—C64—H64A	105.9
C8—C14—H14A	106.2	C65—C64—H64A	105.9
C13—C14—H14A	106.2	C63—C64—H64A	105.9
C15—C14—H14A	106.2	C66—C65—C64	101.9 (4)
C16—C15—C14	101.7 (4)	C66—C65—H65A	111.4
C16—C15—H15A	111.4	C64—C65—H65A	111.4
C14—C15—H15A	111.4	C66—C65—H65B	111.4
C16—C15—H15B	111.4	C64—C65—H65B	111.4
C14—C15—H15B	111.4	H65A—C65—H65B	109.2
H15A—C15—H15B	109.3	O72—C66—C65	111.9 (4)
O22—C16—C15	112.8 (4)	O72—C66—C67	104.9 (4)
O22—C16—C17	104.5 (4)	C65—C66—C67	108.5 (4)
C15—C16—C17	108.3 (4)	O72—C66—H66A	110.5
O22—C16—H16A	110.4	C65—C66—H66A	110.5
C15—C16—H16A	110.4	C67—C66—H66A	110.5
C17—C16—H16A	110.4	C70—C67—C66	104.3 (4)
C20—C17—C16	104.8 (4)	C70—C67—C63	120.3 (4)
C20—C17—C13	120.0 (4)	C66—C67—C63	104.1 (4)
C16—C17—C13	103.8 (4)	C70—C67—H67A	109.2
C20—C17—H17A	109.2	C66—C67—H67A	109.2
C16—C17—H17A	109.2	C63—C67—H67A	109.2
C13—C17—H17A	109.2	C63—C68—H68A	109.5
C13—C18—H18A	109.5	C63—C68—H68B	109.5
C13—C18—H18B	109.5	H68A—C68—H68B	109.5
H18A—C18—H18B	109.5	C63—C68—H68C	109.5
C13—C18—H18C	109.5	H68A—C68—H68C	109.5
H18A—C18—H18C	109.5	H68B—C68—H68C	109.5
H18B—C18—H18C	109.5	C60—C69—H69A	109.5
C10—C19—H19A	109.5	C60—C69—H69B	109.5
C10—C19—H19B	109.5	H69A—C69—H69B	109.5
H19A—C19—H19B	109.5	C60—C69—H69C	109.5
C10—C19—H19C	109.5	H69A—C69—H69C	109.5
H19A—C19—H19C	109.5	H69B—C69—H69C	109.5
H19B—C19—H19C	109.5	C72—C70—C71	116.0 (4)
C22—C20—C21	115.5 (5)	C72—C70—C67	102.5 (4)
C22—C20—C17	103.2 (4)	C71—C70—C67	114.9 (5)
C21—C20—C17	115.0 (5)	C72—C70—H70A	107.7
C22—C20—H20A	107.5	C71—C70—H70A	107.7
C21—C20—H20A	107.5	C67—C70—H70A	107.7
C17—C20—H20A	107.5	C70—C71—H71A	109.5
C20—C21—H21A	109.5	C70—C71—H71B	109.5
C20—C21—H21B	109.5	H71A—C71—H71B	109.5
H21A—C21—H21B	109.5	C70—C71—H71C	109.5
C20—C21—H21C	109.5	H71A—C71—H71C	109.5
H21A—C21—H21C	109.5	H71B—C71—H71C	109.5
H21B—C21—H21C	109.5	O72—C72—O76	110.9 (4)
O22—C22—O26	109.9 (5)	O72—C72—C70	106.3 (4)
O22—C22—C20	105.4 (4)	O76—C72—C70	107.4 (5)
O26—C22—C20	107.8 (5)	O72—C72—C73	107.9 (5)
O22—C22—C23	107.8 (5)	O76—C72—C73	108.9 (4)
O26—C22—C23	109.9 (4)	C70—C72—C73	115.3 (5)
C20—C22—C23	115.7 (5)	C72—O72—C66	105.2 (4)
C22—O22—C16	106.2 (4)	C74—C73—C72	111.5 (5)
C24—C23—C22	111.6 (5)	C74—C73—H73A	109.3
C24—C23—H23A	109.3	C72—C73—H73A	109.3
C22—C23—H23A	109.3	C74—C73—H73B	109.3
C24—C23—H23B	109.3	C72—C73—H73B	109.3
C22—C23—H23B	109.3	H73A—C73—H73B	108.0
H23A—C23—H23B	108.0	C73—C74—C75	110.6 (5)
C23—C24—C25	111.1 (5)	C73—C74—H74A	109.5
C23—C24—H24A	109.4	C75—C74—H74A	109.5
C25—C24—H24A	109.4	C73—C74—H74B	109.5
C23—C24—H24B	109.4	C75—C74—H74B	109.5
C25—C24—H24B	109.4	H74A—C74—H74B	108.1
H24A—C24—H24B	108.0	C77—C75—C76	110.8 (5)
C27—C25—C26	109.6 (6)	C77—C75—C74	111.8 (5)
C27—C25—C24	111.9 (6)	C76—C75—C74	107.4 (4)
C26—C25—C24	107.8 (4)	C77—C75—H75A	108.9
C27—C25—H25A	109.2	C76—C75—H75A	108.9
C26—C25—H25A	109.2	C74—C75—H75A	108.9
C24—C25—H25A	109.2	O76—C76—C75	112.4 (5)
O26—C26—C25	112.0 (5)	O76—C76—H76A	109.1
O26—C26—H26A	109.2	C75—C76—H76A	109.1
C25—C26—H26A	109.2	O76—C76—H76B	109.1
O26—C26—H26B	109.2	C75—C76—H76B	109.1
C25—C26—H26B	109.2	H76A—C76—H76B	107.9
H26A—C26—H26B	107.9	C76—O76—C72	113.0 (4)
C26—O26—C22	113.9 (4)	C75—C77—H77A	109.5
C25—C27—H27A	109.5	C75—C77—H77B	109.5
C25—C27—H27B	109.5	H77A—C77—H77B	109.5
H27A—C27—H27B	109.5	C75—C77—H77C	109.5
C25—C27—H27C	109.5	H77A—C77—H77C	109.5
H27A—C27—H27C	109.5	H77B—C77—H77C	109.5
H27B—C27—H27C	109.5	H1W—O1W—H2W	100 (2)
C52—C51—C60	115.3 (5)		
			
C10—C1—C2—C3	−57.4 (6)	C60—C51—C52—C53	−54.7 (7)
C1—C2—C3—O3	177.4 (4)	C51—C52—C53—O53	−180.0 (5)
C1—C2—C3—C4	57.1 (6)	C51—C52—C53—C54	59.7 (6)
O3—C3—C4—C5	−173.9 (4)	O53—C53—C54—C55	177.9 (5)
C2—C3—C4—C5	−55.0 (6)	C52—C53—C54—C55	−58.9 (6)
C3—C4—C5—C6	−128.5 (5)	C53—C54—C55—C56	−124.0 (5)
C3—C4—C5—C10	53.2 (6)	C53—C54—C55—C60	53.6 (6)
C10—C5—C6—C7	1.6 (8)	C54—C55—C56—C57	176.5 (5)
C4—C5—C6—C7	−176.5 (5)	C60—C55—C56—C57	−0.9 (8)
C5—C6—C7—C8	14.4 (7)	C55—C56—C57—C58	17.8 (8)
C6—C7—C8—C14	−167.1 (4)	C56—C57—C58—C64	−166.3 (4)
C6—C7—C8—C9	−45.3 (5)	C56—C57—C58—C59	−45.5 (6)
C14—C8—C9—C11	−46.5 (6)	C64—C58—C59—C61	−50.3 (5)
C7—C8—C9—C11	−169.2 (4)	C57—C58—C59—C61	−171.4 (4)
C14—C8—C9—C10	−174.8 (4)	C64—C58—C59—C60	−179.4 (4)
C7—C8—C9—C10	62.6 (5)	C57—C58—C59—C60	59.5 (6)
C6—C5—C10—C19	−107.5 (6)	C56—C55—C60—C69	−109.3 (6)
C4—C5—C10—C19	70.7 (6)	C54—C55—C60—C69	73.1 (6)
C6—C5—C10—C1	132.5 (5)	C56—C55—C60—C59	12.9 (7)
C4—C5—C10—C1	−49.3 (6)	C54—C55—C60—C59	−164.6 (5)
C6—C5—C10—C9	14.4 (7)	C56—C55—C60—C51	132.5 (5)
C4—C5—C10—C9	−167.3 (4)	C54—C55—C60—C51	−45.0 (6)
C2—C1—C10—C5	51.3 (6)	C58—C59—C60—C55	−42.3 (6)
C2—C1—C10—C19	−67.8 (6)	C61—C59—C60—C55	−170.9 (4)
C2—C1—C10—C9	170.2 (4)	C58—C59—C60—C69	78.0 (6)
C11—C9—C10—C5	−174.5 (4)	C61—C59—C60—C69	−50.7 (6)
C8—C9—C10—C5	−46.0 (5)	C58—C59—C60—C51	−161.8 (4)
C11—C9—C10—C19	−53.9 (6)	C61—C59—C60—C51	69.6 (5)
C8—C9—C10—C19	74.5 (5)	C52—C51—C60—C55	45.3 (6)
C11—C9—C10—C1	67.5 (5)	C52—C51—C60—C69	−72.2 (6)
C8—C9—C10—C1	−164.0 (4)	C52—C51—C60—C59	166.0 (5)
C8—C9—C11—C12	47.6 (6)	C58—C59—C61—C62	51.2 (6)
C10—C9—C11—C12	174.9 (4)	C60—C59—C61—C62	−179.8 (5)
C9—C11—C12—C13	−54.6 (6)	C59—C61—C62—C63	−54.8 (6)
C11—C12—C13—C14	59.4 (6)	C61—C62—C63—C68	−65.3 (6)
C11—C12—C13—C18	−62.7 (6)	C61—C62—C63—C64	56.2 (6)
C11—C12—C13—C17	170.2 (5)	C61—C62—C63—C67	167.4 (5)
C7—C8—C14—C13	176.9 (4)	C57—C58—C64—C65	−58.7 (6)
C9—C8—C14—C13	55.7 (5)	C59—C58—C64—C65	−179.4 (4)
C7—C8—C14—C15	−59.6 (6)	C57—C58—C64—C63	176.9 (4)
C9—C8—C14—C15	179.3 (4)	C59—C58—C64—C63	56.3 (5)
C12—C13—C14—C8	−61.9 (5)	C62—C63—C64—C58	−59.0 (6)
C18—C13—C14—C8	59.6 (6)	C68—C63—C64—C58	62.3 (6)
C17—C13—C14—C8	178.0 (4)	C67—C63—C64—C58	179.2 (4)
C12—C13—C14—C15	166.5 (4)	C62—C63—C64—C65	168.6 (4)
C18—C13—C14—C15	−72.0 (5)	C68—C63—C64—C65	−70.2 (5)
C17—C13—C14—C15	46.4 (5)	C67—C63—C64—C65	46.7 (5)
C8—C14—C15—C16	−170.0 (4)	C58—C64—C65—C66	−170.2 (4)
C13—C14—C15—C16	−41.4 (5)	C63—C64—C65—C66	−40.8 (5)
C14—C15—C16—O22	135.3 (4)	C64—C65—C66—O72	134.0 (4)
C14—C15—C16—C17	20.2 (5)	C64—C65—C66—C67	18.8 (6)
O22—C16—C17—C20	14.0 (5)	O72—C66—C67—C70	16.9 (5)
C15—C16—C17—C20	134.4 (4)	C65—C66—C67—C70	136.6 (4)
O22—C16—C17—C13	−112.7 (4)	O72—C66—C67—C63	−110.1 (4)
C15—C16—C17—C13	7.7 (5)	C65—C66—C67—C63	9.7 (6)
C14—C13—C17—C20	−148.9 (5)	C62—C63—C67—C70	94.5 (6)
C12—C13—C17—C20	96.8 (6)	C68—C63—C67—C70	−32.8 (7)
C18—C13—C17—C20	−30.1 (7)	C64—C63—C67—C70	−150.0 (5)
C14—C13—C17—C16	−32.5 (5)	C62—C63—C67—C66	−149.2 (5)
C12—C13—C17—C16	−146.8 (4)	C68—C63—C67—C66	83.4 (5)
C18—C13—C17—C16	86.3 (5)	C64—C63—C67—C66	−33.7 (5)
C16—C17—C20—C22	9.7 (5)	C66—C67—C70—C72	7.3 (5)
C13—C17—C20—C22	125.6 (5)	C63—C67—C70—C72	123.4 (5)
C16—C17—C20—C21	136.4 (5)	C66—C67—C70—C71	134.0 (5)
C13—C17—C20—C21	−107.7 (6)	C63—C67—C70—C71	−109.9 (5)
C21—C20—C22—O22	−157.4 (5)	C71—C70—C72—O72	−156.0 (5)
C17—C20—C22—O22	−30.9 (5)	C67—C70—C72—O72	−30.1 (5)
C21—C20—C22—O26	−39.9 (7)	C71—C70—C72—O76	−37.2 (6)
C17—C20—C22—O26	86.5 (5)	C67—C70—C72—O76	88.7 (5)
C21—C20—C22—C23	83.6 (6)	C71—C70—C72—C73	84.4 (6)
C17—C20—C22—C23	−150.0 (4)	C67—C70—C72—C73	−149.6 (4)
O26—C22—O22—C16	−74.3 (5)	O76—C72—O72—C66	−74.0 (5)
C20—C22—O22—C16	41.7 (5)	C70—C72—O72—C66	42.5 (5)
C23—C22—O22—C16	165.9 (4)	C73—C72—O72—C66	166.8 (4)
C15—C16—O22—C22	−151.7 (4)	C65—C66—O72—C72	−154.1 (4)
C17—C16—O22—C22	−34.4 (5)	C67—C66—O72—C72	−36.6 (5)
O22—C22—C23—C24	66.6 (6)	O72—C72—C73—C74	64.8 (6)
O26—C22—C23—C24	−53.2 (7)	O76—C72—C73—C74	−55.7 (6)
C20—C22—C23—C24	−175.7 (5)	C70—C72—C73—C74	−176.6 (5)
C22—C23—C24—C25	53.1 (7)	C72—C73—C74—C75	54.9 (6)
C23—C24—C25—C27	−173.9 (5)	C73—C74—C75—C77	−175.4 (5)
C23—C24—C25—C26	−53.4 (7)	C73—C74—C75—C76	−53.6 (6)
C27—C25—C26—O26	178.7 (5)	C77—C75—C76—O76	179.3 (5)
C24—C25—C26—O26	56.7 (6)	C74—C75—C76—O76	56.9 (6)
C25—C26—O26—C22	−61.2 (6)	C75—C76—O76—C72	−61.9 (6)
O22—C22—O26—C26	−60.9 (5)	O72—C72—O76—C76	−59.6 (5)
C20—C22—O26—C26	−175.4 (4)	C70—C72—O76—C76	−175.4 (4)
C23—C22—O26—C26	57.6 (6)	C73—C72—O76—C76	59.0 (6)

### Hydrogen-bond geometry (Å, º)

**Table:**

*D*—H···*A*	*D*—H	H···*A*	*D*···*A*	*D*—H···*A*
O1*W*—H2*W*···O3^i^	0.87 (2)	2.01 (2)	2.873 (6)	175 (6)
O1*W*—H1*W*···O53^i^	0.87 (2)	2.17 (3)	3.028 (6)	169 (5)
O3—H3···O1*W*	0.90 (7)	1.93 (7)	2.812 (6)	167 (6)
O53—H53···O3^i^	0.93 (7)	2.00 (7)	2.881 (6)	158 (6)

Symmetry code: (i) *x*+1/2, −*y*+3/2, −*z*.
